# Differential reperfusion patterns in retinal vascular plexuses following increase in intraocular pressure an OCT angiography study

**DOI:** 10.1038/s41598-020-73585-0

**Published:** 2020-10-05

**Authors:** Chui Ming Gemmy Cheung, Kelvin Yi Chong Teo, Sai Bo Bo Tun, Joanna Marie Busoy, Amutha Barathi Veluchamy, Richard F. Spaide

**Affiliations:** 1 Singapore Eye Research Institute, Singapore National Eye Centre, 11 Third Hospital Ave, Singapore, 168751 Singapore; 2 Ophthalmology and Visual Sciences Academic Clinical Program (Eye ACP), Duke-NUS Medical School, Singapore, Singapore; 3 University of Sydney, Sydney, Australia; 4 Vitreous, Macula, Retina Consultants, New York, USA

**Keywords:** Diseases, Medical research, Pathogenesis, Optics and photonics

## Abstract

To describe patterns of reperfusion in the superficial vascular plexus (SVP), deep capillary plexus (DCP) and choriocapillaris (CC) as detected on optical coherence tomography (OCTA) in cynomogulus macaque monkey model following increase in intraocular pressure by an intravitreal injection. Animal imaging study. Two cynomogulus macaque monkeys. A 100 µL intravitreal injection (IVI) of saline was given in one eye of each monkey. Serial OCTA using a Zeiss Plex Elite 9000 was used to evaluate reperfusion patterns within the SCP, DCP, and CC. OCTA evidence of perfusion. Pulsation of the central retinal artery was detected after the intraocular pressure was elevated to 98 and ≥ 99 mmHg from IVI. Episodic flow within the SVP arterioles and venules and poor visualization of flow in capillaries was noted during the initial phase of elevated pressure. As the pressure declined, the flow signal within the DCP appeared initially as dots, which progressed laterally to loops which form capillary vortex configuration. Recovery of flow within the SVP and CC appeared sooner than in the DCP. At 40 min after the injection, well after the intraocular pressure normalized, the retinal and choriocapillaris vascular perfusion showed focal defects in every layer. Compared with pre-injection images, vessel density in the DCP was 68.8% and 78.6% of baseline in monkey 1 and monkey 2, respectively. In contrast vessel density in the SVP recovered to 84.2% and 88.9% of baseline. Increases in intraocular pressure from IVI have the potential to affect every layer of blood flow in the fundus. After nominal return of intraocular pressure, focal defects in flow persisted, which may result in longer term damage to the retina.

## Introduction

Intravitreal injection (IVI) is one of the most performed ophthalmic interventions since the advent of vascular endothelial growth factor (VEGF) inhibitors as standard of care for neovascular age-related macular degeneration, diabetic macular edema and retinal vein occlusion. The number of intravitreal injections has been rapidly increasing from 5000 IVI annually worldwide between 1997–2001 to an estimated 5.9 million administered in 2016 in the United States alone^1^. Increased intraocular pressure with IVI remains a concern despite a generally favourable safety profile. While IOP rise post-IVI generally tend to be transient, there is still concern that repeated episodes may be a cause or exasperating factor for glaucoma progression^2,3^. Pressure related ocular damage may be directly caused by pressure itself, or by indirect effects on tissue perfusion related to the elevated intraocular pressure.

Fluorescein angiography has been traditionally used to evaluate the vasculature of the retina but this modality principally evaluates the superficial vascular plexus^4,5^. A newer modality, OCTA, enables non-invasive depth-resolved study of the vasculature of the eye. These layers can be evaluated by OCTA and are divided into the superficial vascular plexus (SCP), which invests the ganglion cell layer^6^, the intermediate vascular plexus and the deep vascular plexus (DCP) of which both bracket the inner plexiform layer^4,7^. Frame averaging of OCTA improves the clarity of vascular imaging, and offers the possibility of in vivo studies of flow disturbance in response to increase in IOP^8^.

In the current study, we aim to investigate the effects of increased IOP related to IVI on the perfusion of SVP, DVP and CC as detected on OCTA in a cynomolgus macaque monkey model.

## Methods

All procedures were carried out in the SingHealth Experimental Medicine Centre, which is licensed by the Agri-Food and Veterinary Authority (AVA) of Singapore and is fully accredited by the Association for Assessment and Accreditation of Laboratory Animal Care International (AAALAC). The study adhered to the Association for Research in Vision and Ophthalmology (ARVO) Statement for the Use of Animals in Ophthalmic and Vision Research and the National Advisory Committee for Laboratory Animal Research (NACLAR) guidelines in Singapore. Ethical approval was obtained from the SingHealth Institutional Animal Care and Use Committee (IACUC) (Approval Reference Number: 2018/SHS/1443).

### Animals and protocol

Two adult male cynomolgus macaque monkeys (*Macaca fascicularis*), (estimated age 12–14 years, weight 3–6 kg) were included into the study. Before recruitment, all animals were given a comprehensive ocular examination, including of the anterior segment and fundus, to exclude ocular disease. All slit-lamp examination and imaging were performed under general anaesthesia, involving intramuscular (IM) ketamine hydrochloride (20 mg/kg), IM acepromazine maleate (0.25 mg/kg) and IM atropine sulfate (0.125 mg/kg).

After pupil dilation with 1% tropicamide and anesthesia with topical proxymetacaine (Proparakain-pos 0.5%; Ursapharm), intraocular pressure was measured with Tonoshield (highest registrable reading 99mHg). Blood pressure (BP) and other vital signs were performed by NIBP built-in with the Dre Waveline VS Veterinary Monitor. Mean arterial pressure (MAP) and mean ocular perfusion pressure (MOPP) were calculated based on the formulae MAP = Diastolic BP + 1/3(systolic-diastolic BP) and MOPP = 2/3(MAP)-IOP^9^. Baseline OCT angiography (PlexElite, Carl-Zeiss, Germany) of 3 mm × 3 mm centered over the fovea was performed in both eyes of each animal. Indocyanine green angiography (ICGA) was performed with the Spectralis; Heidelberg Engineering, Heidelberg, Germany. IV injection of Indocyanine Green (0.75 mg/kg) via the saphenous vein were performed.

One eye of each animal was given a 100 µL intravitreal (IVT) injection of saline through the pars plana. Intraocular pressure post-IVT was documented. Serial OCTA was performed in the injected eye and repeated every 30 s up to 7-min post-injection. A final set of OCTA was performed at 40-min post-injection.

OCTA images were acquired by trained technicians. We only included images with a signal strength of nine and above. We used the preset segmentation boundaries of the PlexElite system for the superficial (ILM to IPL) and deep retinal plexuses (IPL to OPL). (This strategy includes the amalgamated imaging of the intermediate capillary plexus with the DCP^6^. Use of the term DCP in this article acknowledges this amalgamation.) For segmentation of the choriocapillaris, we selected a slab with boundaries 17 µm below RPE to 33 µm below the RPE. This was based on previous imaging of the choriocapillaris in monkeys in which image averaging was used on various depths in the inner choroid. The 17–33 µm slab below the RPE produced the best image quality of the choriocapillaris with the least artefacts. Manual adjustment of the segmentation was performed when the automated segmentation was seen to be inaccurate. The SVP, DCP, and CC slabs were entered into a red–green–blue (RGB) color stack. Once the image sandwiches are made, as done by a macro, the images are aligned in a 3-step process using a FIJI a distribution of the program FIJI with the plugin Register Virtual Stack Slices (https://imagej.net/Register_Virtual_Stack_Slices). This plugin can use several different forms of feature extraction from any given image and then use one of several different methods of registration. The approach used in the current method was one of successive approximation. A rigid model was first used for gross image registration and the non-overlapping portions of the images (caused by variations in fixation) were cropped from the image. Then a least squares registration was done followed by an affine b-spline approach^8^ to fine tune the registration.

### Quantification of vessel density

Image J was used for quantification of vessel density (VD) as used in previous published studies^10,11^. Briefly, images of the SCP and DCP were binarized by the Otsu method (Specification of this algorithm can be found online https://imagej.net/Auto_Threshold). This method is a global thresholding algorithm for image binarization. Optimal threshold value was calculated and evaluated by between-class variance (or within-class variance). Binarized images underwent further attribute filtering (Gray Scale Attribute Filtering^12^ with opening attribute set a minimum area of 5 and connectivity at 4) to remove noise and outliers. The VD measure was expressed as the ratio of white to black pixels within the 3 × 3 mm imaged area.

## Results

### Monkey 1

**Figure 1:**
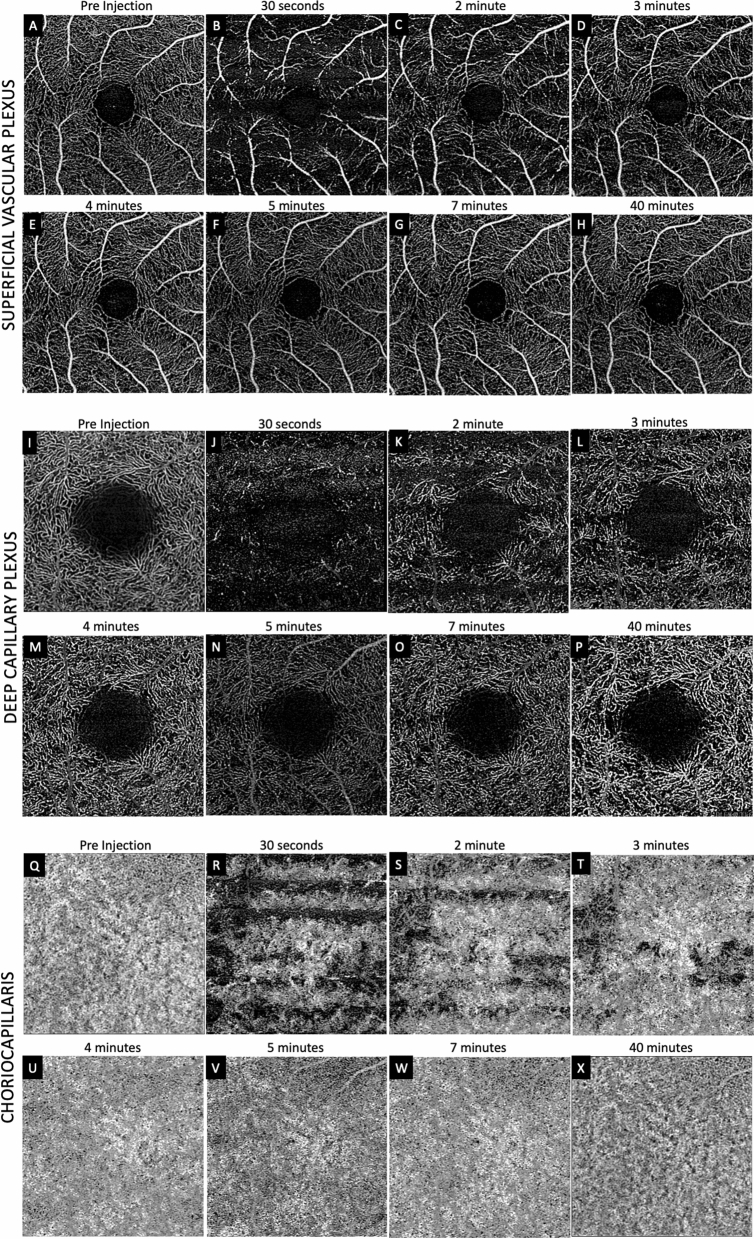
Sequential optical coherence tomography angiography (OCTA) Monkey 1. (**A–H**) shows superficial vascular plexus (SVP), deep capillary plexus (DCP) and choriocapillaris (CC) layers. Panel (**A**), (**I**) and (**Q**) show pre-injection OCTA of SVP, DCP and CC respectively. At 30 s post injection, when intra ocular pressure (IOP) was 98 mmHg, flow was only noted in main arterioles and veins with minimal detection of flow signal within the capillary beds in the SVP (**B**). At the same time, there was marked reduction in flow signal in the DCP (**J**) and in the CC (**R**). Dark horizontal bands are observed in all three layers. Progressively more flow signal was noted over time in the SVP (**C**–**G**), DCP (**K**–**O**) and CC (**S**–**W**). By 7 min, when IOP was 22 mmHg. As flow signal increased in the DCP, recognizable capillary units arranged around an epicenter could be appreciated (**L**–**O**), despite persistence of patchy areas of flow deficit. In the CC, areas of flow deficit progressively decreased (**R**–**U**). By 40 min (**H**, **P** and **X**) the flow distribution in the SVP and CC resembled the respective pre-injection images, but flow deficits in the DCP were more evident when compared to the pre injection image.

**Figure 2:**
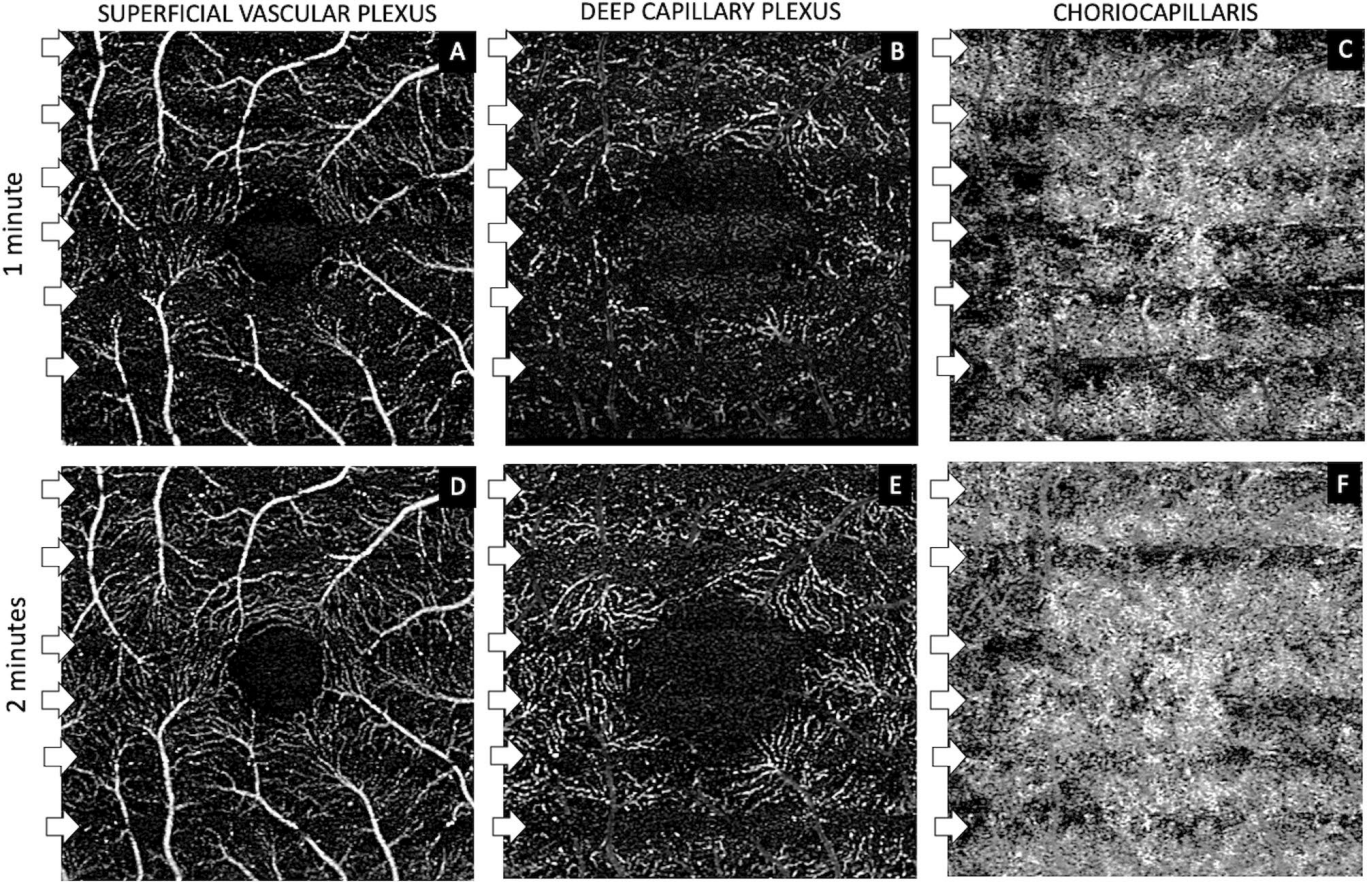
Stripe dark band patterns across OCTA scans at 1-min and 2-min post-intravitreal injection. Evenly distributed horizontal stripe patterns (white arrows) across each OCTA scan at the superficial vascular plexus (SVP) (**A**, **D**), deep capillary plexus (DCP) (**B**, **E**) and choriocapillaris (CC) layers (**C**, **F**) was detected. These bands represent episodic flow signal as the intra ocular pressure (IOP) dropped below the instantaneous pressure in the arteries and veins. These 6–7 bands persisted for about 2–3 min post injection and if timed according to the duration of scan (approximately 3 s) would imply a heart rate of approximately 120 beats per minute, which was in agreement with the anesthesia record (101–128 beats per minute). These bands were more prominent and persistent in the DCP (**B**, **E**) than in the SVP (**A**, **D**) suggesting a lower perfusion pressure in the DCP.

**Figure 3:**
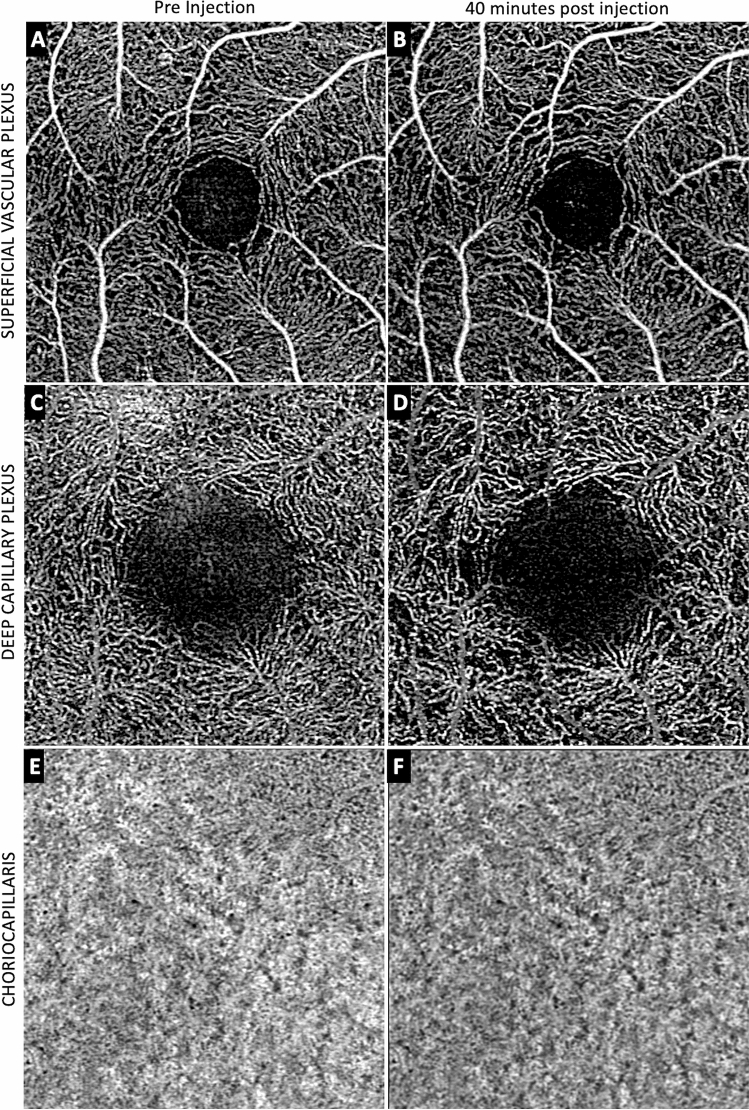
Averaged OCTA images of pre- and 40-min post-injection of each retina layer of Monkey 1. Panel (**A**), (**C**) and (**E**) showed the OCTA images of the superficial vascular plexus (SVP), deep capillary plexus (DCP) and choriocapillaris (CC) layers pre injection and the corresponding 40-min post-injection images (**B**, **D**, **F**). In the SVP, the 40-min post injection image (**B**) is very similar to the pre-injection image (**A**) with only subtle and minimal areas of flow deficit compared to pre-injection. In the CC layer, the 40-min post-injection image was also similar to the pre-injection image (**E**, **F**) suggesting almost complete recovery of perfusion. The DCP however, showed persistent areas of reduction in flow in the 40-min post injection image (**D**) compared to pre-injection (**C**).

Serial OCTA of the SCP, DCP and CC were performed pre-IVT and post-IVT up to 40 min (Figs. 1, 2, 3). Intraocular pressure before IVT was 9 mmHg and rose to 98 mmHg immediately following IVT. Pulsation of the central retinal artery was visualized. By 7 min, IOP had returned to 22 mmHg. A late post-IVT image was acquired 40 min after IVT. Approximately six evenly distributed dark bands, or a striped pattern, of similar height were observed in each of the SVP, DCP and CC slabs in the images obtained up to 2 min post-IVI (Fig. 2). The bands became less obvious in the SVP by 2 min but persisted in the DCP and CC up to 3 min.

In the SVP, alternating long arterioles and venules, connected by transverse capillaries forming an interconnected plexus can be seen in the pre-IVI OCTA (Fig. 1A). These correlated with retina arteries and veins on ICGA. At 30 s post-IVI, flow signal was evident in the main arterioles and veins, while only faint flow signal was detected within the capillary bed (Fig. 1B). By 2 min post-IVI, flow signal was detected in both arterioles, venules, first degree branches and the outline of the terminal capillary ring surrounding the FAZ became visible (Fig. 1D). Over the next minute, there was lateral progression in the flow signal within the capillary bed and more flow signal within arteriole-to-venule communications are evident. From 4-min post-IVI onwards, the details of the SVP started to resemble that at pre-IVI (Fig. 1D–G). At 40 min post-IVI when IOP was 14 mmHg, the details of the SVP were comparable to pre-IVI image, although a few isolated areas of non-perfusion can still be seen (Fig. 1, 3).

In the DCP, capillary beds arranged in polygonal patterns converging on central loci can be seen in the pre-IVI image (Fig. 1I). At 30 s post-IVI, only a few dots were visible and there was marked reduction in flow signal across the entire OCTA image (Fig. 1J). At 2 min post-IVI, flow signal has progressed to more segments of vessels beyond the dots (Fig. 1K). Over the next few minutes, lateral progression of flow signal developed into recognizable capillary units arranged around an epicenter (Fig. 1L–O). When the 40 min post-IVI image was compared to the pre-IVI image, however, patchy areas of residual non-perfused capillary bed was still detected throughout the macula (Fig. 1, 3).

In the CC, a uniform meshwork of fine vessels was observed in the pre-IVI image (Fig. 1Q). After IVI, large patches of non-perfusion were evident (Fig. 1R–U) and these occurred with the same striped pattern seen in the retinal vascular layers (Fig. 2). These areas became progressively smaller over the next few minutes. At 40 min post-IVI, flow signal in the CC was comparable to the pre-IVI image (Fig. 1, 3).

### Monkey 2

**Figure 4:**
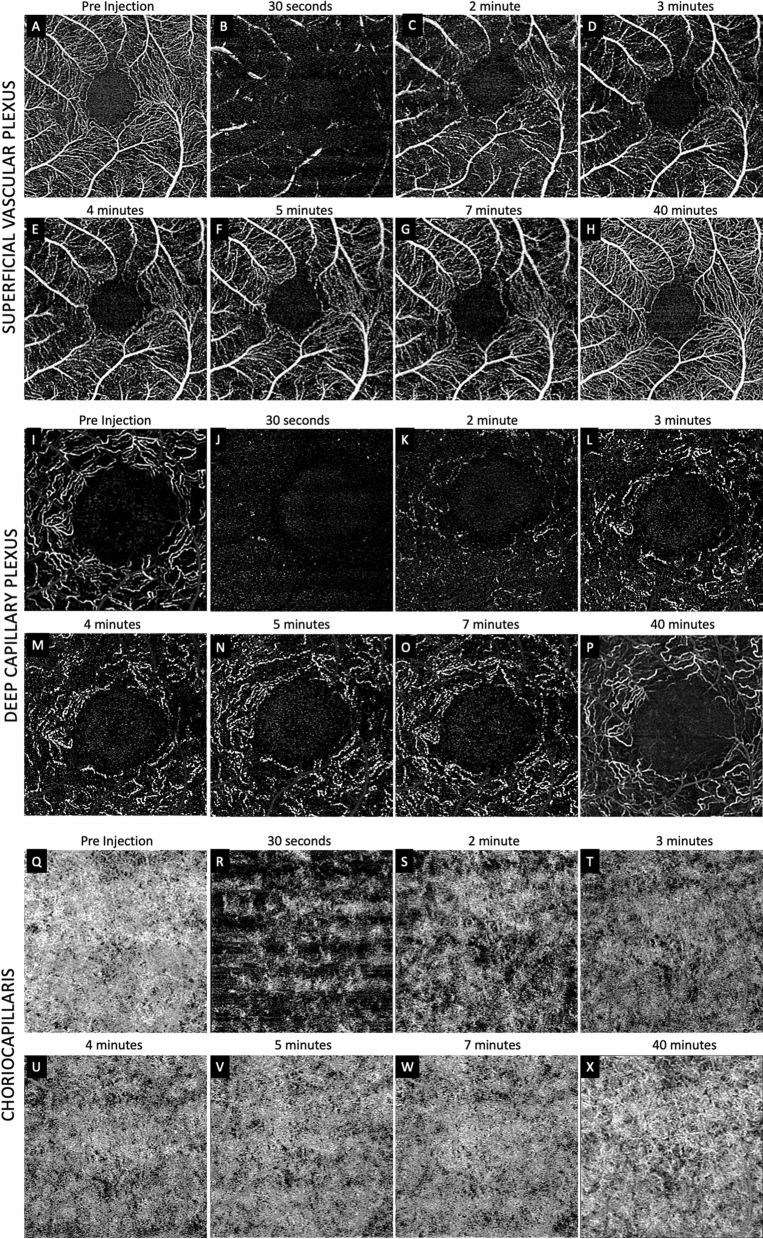
Sequential optical coherence tomography angiography (OCTA) Monkey 2. (**A**–**H**) shows superficial vascular plexus (SVP), deep capillary plexus (DCP) and choriocapillaris (CC) layers. Panel (**A**), (**I**) and (**Q**) shows pre injections OCTA of SVP, DCP and CC respectively. Intra ocular pressure (IOP) was 99 mmHg at 30 s which dropped to 14 mmHg at 7 min. The progression of flow throughout the layers were similar to Monkey 1, but the delay in reperfusion was more marked in Monkey 2. At 30 s, minimal to no flow was detected within the capillary beds and none in the venules in the SVP (**B**) as compared to Monkey 1 at the same time point. This difference was even more evident when comparing the DCP between animals where recognizable capillary units were only detected after 3–4 min post-injection (**M**, **N**, **O**). There were more evident areas of persistent flow deficits in the CC at 40-min post-injection (**X**) as compared to Monkey 1. Defects in perfusion was also most apparent in the DCP (**P**) compared to the SVP (**H**) and CC at 40 min.

Serial OCTA of the SVP, DCP and CC were performed up to 40 min post-IVI (Fig. 4). Intraocular pressure before IVI was 15 mmHg and rose to ≥ 99 mmHg immediately following IVI. Pulsation of the central retinal artery was visualized. By 7 min, IOP had returned to 14 mmHg. A late post-IVI image was acquired at 40 min after IVI. Episodic recovery of blood flow was observed during the first 2–3 min post-IVI. Horizontal dark bands similar to Monkey 1 were observed in all layers during the first 2–3 min post-IVI.

The pattern of flow recovery in the SCP, DCP and CC were similar to those observed in the first monkey (Fig. 4). However, the pre-injection images, especially of the DCP and CC suggest a less healthy vasculature compared to monkey 1 (Pre injection SVP-VD: 42.3% and 38.1% in monkey 1 and 2 respectively and pre injection DCP-VD: 41.7% versus 12.2% in monkey 1 and 2 respectively) (Fig. 4A, I, Q). After IVI, the delay in reperfusion appears to be more marked in Monkey 2 compared to Monkey 1. At 30 s post-IVI, flow signal was only detected in the arterioles but not the venules or capillary bed in the SVP (Fig. 4B), and only a few dots were visible in the DCP (Fig. 4J). In the DCP, patchy capillary units were barely recognizable around 4-min post-IVI (Fig. 4M,N).

At 40 min after the injection, IOP was < 21 mmHg. The retinal and CC vascular perfusion showed focal defects as compared with the pre-injection images, which were more pronounced in the DCP than the SVP.

In both animals, the reduction in vessel density was more pronounced in the DCP than the SVP. At 2 min post-injection VD was reduced to 61.2% and 50.1% (SVP) and 46.8% and 35.2% (DCP) of pre injection VD in monkey 1 and monkey 2, respectively. At 40 min, the reduction was to 88.9% and 84.2% (SVP) and 78.6% and 68.6% (DCP) of pre injection VD in monkey 1 and monkey 2, respectively. We did not observe and hyperreflectivity developing in the cross sectional or enface OCT in either monkey that may suggest paracentral acute middle maculopathy. During the imaging session the heart rate was recorded as 101–128 beats per minute.

## Discussion

In the current study, we found increased IOP affected the three main layers evaluated, the SVP, DCP, and the CC. Of these the DCP seemed to be affected most. In the SVP, flow signal appeared in the large arterioles and venules and their first order branches within 30 s. There was suggestion of some flow signals from capillaries in the SVP by 30 s. By 2 min the perifoveal vascular ring was visualized in the SVP slab. Given this is partially a segmentation issue^13^, the flow signal in the DCP remained patchy and incomplete. At 40 min post-IVT, well after IOP had returned to normal range, the flow signal in the SVP had almost recovered to pre-IVI status, but deficit was still observed within the DCP, and to a lesser extent in the CC. These findings have several important implications.

The OCTA findings can be interpreted with consideration of the mismatch between IOP and mean arterial pressure (MAP), which determine the mean ocular perfusion pressure (MOPP) at the central retinal artery (Table 1). At baseline, MOPP was 36.4 mmHg in both monkeys. Immediately post-IVI, this dropped to < 0 mmHg. The central retinal artery (CRA) was temporarily occluded. Over the next 30 s, flow within the CRA started to return with pulsation visible, suggesting the IOP had dropped to between systolic and diastolic blood pressure. Flow was only detected in the large retinal arterioles and venules, but not in the capillary bed of the SVP as the flow rate in the large vessels exceeded the threshold for detection. As IOP started to normalize further, episodic flow signal began to appear in the SVP capillary bed, appearing as evenly-spaced horizontal dark bands. The appearance of flow signal indicated IOP had dropped below the instantaneous pressure in the vessels. The monkeys showed about 6 or 7 of the stripes of flow data per image in the periods of high intraocular pressure which persisted for 2–3 min post-IVI. The periods of flow were thought to be related to the animals’ pulse. As the OCTA scan takes approximately 3 s to complete, this would imply the monkey heart rate was approximately 120 per minute, which was in agreement with the anaesthesia record (range 101–128 per minute). The observation that these bands are more prominent and persistent in the DCP than in the SVP suggests that flow rate in the DCP is lower than in the SVP. This observation highlights an important limitation of the OCTA in the ability to image slower flow. A threshold for detection exists for OCTA, below which current OCTA technology cannot detect flow. We may assume that flow below threshold of detection on OCTA is likely below that required for maintenance of healthy tissue.

**Table 1:** Summary of Mean arterial pressure (MAP), Intraocular pressure and Mean ocular perfusion pressure (MOPP) at baseline, immediately post-intravitreal injection (IVT), 7 minutes post-IVT and 40 minutes post-IVT.

	Baseline	Immediately post-IVT	7 min post-IVT	40 min post-IVT
**Monkey 1**				
MAP	63.7	63.3	56.7	54.0
IOP	9	98	22	11
MOPP	33.5	< 0	15.8	25.0
**Monkey 2**				
MAP	69.7	66.0	47.7	43.0
IOP	15	≥ 99	14	10
MOPP	31.5	< 0	17.8	18.7

*MAP* mean arterial pressure (MAP = DBP + 1/3(SBP-DBP), *IOP* intraocular pressure, *MOPP* mean ocular perfusion pressure MOPP = 2/3(MAP)-IOP, *DBP* diastolic blood pressure, *SBP* systolic blood pressure.

Within the first 30 s post-IVI, flow in the DCP was impaired to a much larger extent compared to the SVP. The flow deficit within the DCP also persisted for longer than in the SVP, as demonstrated by the lower percentage of reperfusion compared to baseline in the DCP at both 2 min and 40 min post-IVI. While reduction of systemic blood pressure likely contributed to reduction in MOPP at the central retinal artery, the relative difference between SVP and DCP cannot be explained only by change in systemic blood pressure. We propose that our observation suggests that the perfusion pressure within the DCP is lower than the SVP. In addition, the prompt recovery of flow within the arterioles and venules within the SVP despite prolonged DCP flow deficit suggests the observed flow was from the inner layers of the retina and did not have significant contribution from the DCP.

We observed markedly different organization of the SVP and DCP, which is in keeping with previous studies. Starting from 30-s post-IVI, flow signal within the DCP began in the form of diffusely arranged small dots, which expanded laterally^14,15^. This observation is in keeping with histological studies which showed that the DCP received few connections from the other vascular planes^6^. The arterial supply to the DCP was derived from descending branches of the precapillary artery. The blood flow to the capillaries of the SVP is from the precapillary arterioles. The precapillary arterioles are also the source of blood to the DCP. However, while the resistance of flow in the SVP is only the capillary segment at that point, the total resistance of blood flow for blood in the DCP circuit is the descending arteriole, the DCP itself and the ascending venule. Thus, the perfusion pressure at the level DCP is lower than the capillary segment in the SVP.

Snodderly and Weinhaus stated the venous drainage from the DCP was seen to course for up to several hundred micrometers to meet with a venous tributary from the ganglion cell layer^6^. Histological studies showed vessels from the DCP occasionally could directly anastomose with larger retinal veins^6,16,17^. This suggests the inflow pressure to the DCP is less than the capillary pressure in the SVP. While the SVP capillary is directly fed by the precapillary arteriole, a connecting capillary must conduct blood from the precapillary arteriole to the DCP. The DCP is then distributed into a branching mesh of vessels. The outflow from the DCP then leads to tributary branches or directly to larger retinal veins which expected to have a lower pressure than the postcapillary venules and also drain capillaries in the superficial layers.

In contrast, to the SVP and DCP, the CC derives its blood supply from short posterior ciliary arteries rather than from the central retinal artery. At 40 min, some perfusion defects persisted in the CC, much the same as the other vascular layers. Comparison between the three layers at 40 min post-IVI suggest the most severe persistent flow deficit in the DCP, followed by CC, whereas the SVP showed almost complete recovery.

It is possible the focal defects in perfusion seen in the present series are a short-term manifestation of ischemia/reperfusion injury. Prior animal studies have elucidated the pathophysiologic mechanisms of this injury in the retina. Ischemia leads to VEGF production, which causes ICAM-1 expression and leads to leukocyte plugging in retinal capillaries^18,19^. The leukocytes adhered to the vasculature through CD18 and remodelled it through Fas ligand (FasL)-mediated endothelial cell apoptosis. Anti-VEGF treatment has been shown to mitigate some of the damage from ischemia^20,21^. Thus, the volume of injection of anti-VEGF agents may cause ischemia from increased intraocular pressure, but the pharmacologic effect of the anti-VEGF agent may prevent some of the potential ischemic injury mediated by raised IOP. It remains to be seen if there is cell damage related to recurrent vascular defects, if the vascular defects may fully recover, or whether the retinal vasculature may become increasingly susceptible to irreversible damage with repeated episodes of IOP rise in patients receiving multiple IVI, especially in at-risk groups such as those with glaucoma or pre-existing vasculopathy. Sustained release of anti-VEGF agents may obviate these possibilities.

There are weaknesses of this study to mention, including the small number of animals evaluated and the limited sampling of the inherent variability in monkeys. Even between the two monkeys, notable differences in the retinal vasculature were observed at baseline, with monkey 2, estimated to be 2 years older than monkey 1, having a notably less healthy vasculature. The volume of 100 µL in a monkey eye (axial length ~ 20 mm) is estimated to be equivalent to a 170 µL intravitreal injection in a human eye. Hence, the elevated IOP observed is an exaggerated model compared to the effect of a 50 µL intravitreal injection in clinical practice. In comparison, a previous study in 213 consecutive injections reported mean IOP to rise from 14 to 44 mmHg (range 4–87 mmHg)^22^. Since 99 mmHg was the highest registrable on the Tonoshield, it is possible that even higher IOP was induced immediately after the injection. Despite the elevated IOP, all the OCTA images had signal strength of at least 9 out of 10. As such, it is unlikely that the results are due to media perturbation. The use of anesthesia is known to lower blood pressure. The resultant reduction in MAP likely further exacerbated the effects of raised IOP. However, the differential recovery pattern between the SVP and the DCP cannot be explained purely by the drop in MAP. In particular, it does not explain the focal defects observed. The OCT instrument used does not directly segment the intermediate and deep plexuses. Changes beyond 40 min, and whether full reperfusion may be seen at a later timepoint cannot be addressed with the current data. Nonetheless, this study highlights some possibilities offered by novel non-invasive imaging.

We report differential reperfusion patterns using OCTA to evaluate the SVP, DCP, and CC after a transient pressure rise. These may lead to ischemic damage. Although there are differences in the time course of reperfusion, the sensitivity of retinal cells varies by layer^20^, and the amount of ischemic injury may vary as well. These novel findings may have physiological implications in health and disease involving the DCP. Increases in intraocular pressure from IVI may induce longer lasting abnormalities in flow.
